# Cattle Manure Enhances Methanogens Diversity and Methane Emissions Compared to Swine Manure under Rice Paddy

**DOI:** 10.1371/journal.pone.0113593

**Published:** 2014-12-10

**Authors:** Sang Yoon Kim, Prabhat Pramanik, Paul L. E. Bodelier, Pil Joo Kim

**Affiliations:** 1 Division of Applied Life Science (BK 21 Program), Gyeongsang National University, Jinju, South Korea; 2 Institute of Agriculture and Life Science, Gyeongsang National University, Jinju, South Korea; 3 Netherlands Institute of Ecology (NIOO-KNAW), Department of Microbial Ecology, Wageningen, The Netherlands; Agricultural Research Service, United States of America

## Abstract

Livestock manures are broadly used in agriculture to improve soil quality. However, manure application can increase the availability of organic carbon, thereby facilitating methane (CH_4_) production. Cattle and swine manures are expected to have different CH_4_ emission characteristics in rice paddy soil due to the inherent differences in composition as a result of contrasting diets and digestive physiology between the two livestock types. To compare the effect of ruminant and non-ruminant animal manure applications on CH_4_ emissions and methanogenic archaeal diversity during rice cultivation (June to September, 2009), fresh cattle and swine manures were applied into experimental pots at 0, 20 and 40 Mg fresh weight (FW) ha^−1^ in a greenhouse. Applications of manures significantly enhanced total CH_4_ emissions as compared to chemical fertilization, with cattle manure leading to higher emissions than swine manure. Total organic C contents in cattle (466 g kg^−1^) and swine (460 g kg^−1^) manures were of comparable results. Soil organic C (SOC) contents were also similar between the two manure treatments, but dissolved organic C (DOC) was significantly higher in cattle than swine manure. The *mcr*A gene copy numbers were significantly higher in cattle than swine manure. Diverse groups of methanogens which belong to *Methanomicrobiaceae* were detected only in cattle-manured but not in swine-manured soil. Methanogens were transferred from cattle manure to rice paddy soils through fresh excrement. In conclusion, cattle manure application can significantly increase CH_4_ emissions in rice paddy soil during cultivation, and its pretreatment to suppress methanogenic activity without decreasing rice productivity should be considered.

## Introduction

Methanogens, the phylum *Euryarchaeota* within the domain of the *Archaea*, are important in global C cycle that mineralizes crop residues and soil organic matter under anaerobic conditions. Large amounts of methane (CH_4_) are released to the atmosphere as the end-product of archaeal metabolism [1]. Methanogens are found in a diverse range of habitats, for instance: wetlands, rice fields, fresh and marine water sediments, digestive tracts of ruminants and termites, anaerobic waste digesters, and geothermal vents [2]. Among these habitats, flooded rice paddies are major contributors of CH_4_ to the atmosphere [3] and range between 39 to 112 Tg CH_4_ per year [4]. With increasing demand for rice due to rapid population growth, annual worldwide rice production must increase by 8–10 million tons over the next 20 years [5] which can dramatically increase CH_4_ emissions from flooded rice paddy fields.

Soil organic carbon (SOC) plays an important role in maintaining soil fertility and health and crop production [6]. Livestock manures have been widely used as organic amendments and are important inputs of C and nutrients. For instance, manure application (swine manure-applied at 833 kg ha^−1^) for 18 years enhanced SOC, total nitrogen (N), available N, and available phosphorus (P) contents by 19.2%, 14.4%, 13.2%, and 78.3%, respectively, compared with control (without pig manure) [6]. Manure application also increased soil biological properties by enhancing soil microbial biomass C and N by 48.9% and 33.2%, respectively and eventually improved the rice productivity by 133% over control treatment [7]. However, manure addition can significantly increase CH_4_ emissions due to enhanced rice productivity and nutrient availability in the flooded paddy ecosystems [8], [9]. Livestock manures, such as cattle and swine, may vary in their physicochemical and biological properties [10], which might have variable effect on microbial communities and CH_4_ emissions in rice paddy soils. Many studies have shown that compost or manure applications enhance soil microbial and enzyme activities and diversity [11], [12]. Radl et al. (2007) suggested that specific methanogens from cattle rumen can be transferred to grassland soil through excrements, contributing to CH_4_ production. As most of rice is cultivated under the flooded condition, it may also be a better habitat for rumen-derived methanogens than in upland conditions. Although CH_4_ emissions can be higher in rumen-derived methanogens from cattle-manured soils, there is no information in the literature comparing relative effects of different livestock manures from ruminant and non-ruminant animals on CH_4_ emission and methanogenic microbial communities in rice paddy soils.

In this study we tested the following hypothesis: compared with swine manure, cattle manure can (i) increase methanogenic activity due to higher labile carbon content, and (ii) serve as an inoculum for methanogens in the rice paddy soil. We conducted a greenhouse experiment to study the effects of two different livestock manures [cattle (ruminant) manure and swine (non-ruminant) manure] on CH_4_ emissions and the changes in methanogenic abundance and diversity in rice paddy soil. Our objectives were to compare the effects of cattle and swine manures on CH_4_ emissions and their methanogenic abundance and diversity under rice in the greenhouse.

## Materials and Methods

### Greenhouse experiment

The experiment was conducted in a greenhouse. Soil was collected using a pre-cleaned stainless steel hand shovel from the ploughed layer (0–15 cm) in a typical rice paddy field in the spring of 2009 at Gyeongsang National University Agronomy field in Jinju, South Korea. The soil sample was air-dried, sieved (<10 mm) and packed to 1.2 Mg m^−3^ bulk density (15 kg dried soil) into Wagner pot (0.05 m^2^, 25 cm in diameter and 30 cm in height). The soil belongs to the *Pyeongtaeg* series (fine-silty, mixed, non-acid, mesic Typic Endoaquept; Sand 30%, Silt 55% and Clay 15%) [14]. The soil had pH 7.2±0.1 with following characteristics: SOC, 6.96±0.6 g kg^−1^; dissolved organic C (DOC), 40.5±13.4 mg kg^−1^; available P_2_O_5_, 57.7±2.0 mg kg^−1^ and exchangeable Ca^2+^, Mg^2+^ and K^+^ 5.15±0.20, 0.66±0.03 and 0.11±0.01 cmol^+^ kg^−1^, respectively.

Manure samples were collected from a livestock farm at Gyeongsang National University. The cattle manure contained total organic C, 466±4 g kg^−1^; total N, 18.6±1.5 g kg^−1^; total P_2_O_5_, 8.6±0.5 g kg^−1^; total K_2_O, 1.3±0.1 g kg^−1^; mean C/N ratio, 25.1; DOC 21.4±4.1 g kg^−1^ and water content, 681 g kg^−1^ (mass mass^−1^). In contrast, the swine manure had total organic C, 460±3 g kg^−1^; total N, 14.9±0.7 g kg^−1^; total P_2_O_5_, 9.8±0.3 g kg^−1^; total K_2_O, 1.2±0.1 g kg^−1^; mean C/N ratio 30.9, and DOC 7.9±0.2 g kg^−1^ and water content 677 g kg^−1^ (mass mass^−1^). Cattle and swine manures were applied into the pot a day before transplantation at the rate of 0 (chemical fertilizer alone, control), 20 (recommended), and 40 Mg fresh weight (FW) ha^−1^, which roughly corresponded to 0, 11 and 22 g FW kg^−1^ in the pot experiment, respectively. Chemical fertilizers were applied in the same way in all treatments (including control) with the ratio of N–P_2_O_5_–K_2_O = 110–45–58 kg ha^−1^ by using urea, triple superphosphate and potassium chloride and then mixed with the soil. The recommended dose of livestock manure is approximately 20 Mg ha^−1^ along with chemical fertilizer using the Korean standard rice cultivation guidelines in rice paddy soils maintaining soil quality and rice productivity [15]. The total nutrient inputs from chemical fertilizers and manures were presented in S1 Table. The pots were arranged in a random manner and each treatment was carried out in triplicate.

Three (3) 30 days old rice (*Oryza sativa* L.) seedlings were transplanted on June 15, 2009. The water level was maintained at 5–7 cm above the soil surface by periodical watering as and when required throughout the crop growing season and then drained 2 weeks before rice harvesting. Herbicide and pesticide were not applied to avoid side effects on CH_4_ emissions and methanogens during rice cultivation. The rice was harvested 120 days after transplanting (DAT) and the grain yield was recorded properly following Korean standard rice cultivation guidelines [16]. The whole above-ground biomass was harvested from pots and air-dried in the greenhouse. The grains were separated and weights of straw and grains were measured separately.

### Measurement of CH_4_ emissions

Methane emissions from the rice-planted pots were measured by using the closed chamber method for the entire cropping periods [17]. Transparent poly acrylic plastic chambers (diameter 24 cm, and height 100 cm) equipped with a circulating fan for gas mixing and a thermometer to monitor inside temperature was used during the sampling. The gas samples from the chambers were collected every 3 hr interval during the day to determine the optimum sampling time for sampling. Similar CH_4_ fluxes to the daily mean fluxes were observed between 10∶00 and 13∶00 hrs. Gases were sampled once in a week using 50 ml air-tight plastic syringes at 0, 10, 20, and 30 min intervals after manually closing the chamber. Gas sampling and air temperature measurements were simultaneously carried out. The CH_4_ gas was immediately transferred into a 30 ml pre-evacuated vial sealed with a butyl septum cap. The CH_4_ concentration in the gas sample was measured using gas chromatography [18]. Methane flux was calculated as the increase in CH_4_ concentration per unit surface area of the chamber for specific time interval. The following closed chamber equation was used for estimation of CH_4_ flux from each treatment [17]:
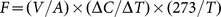
where, F is the CH_4_ flux (mg CH_4_ m^−2^ hr^−1^); V is the volume of the chamber (m^3^); A is the area (m^2^); Δc/Δt is the rate of accumulation of CH_4_ gas concentration in the chamber (mg m^−3^ hr^−1^ for CH_4_) in the time t, and T is the absolute temperature (273+ mean temperature in chamber,°C) during gas sampling.

In detail, linear regression was calculated between the gas concentration and time (0, 10, 20 and 30 min). If one sample deviated from the line, the flux was recalculated without the outlier. If the regression coefficient (r^2^) was less than the 90% confidence limit, the sample was rejected.

The total CH_4_ flux for the entire crop period was calculated by modifying earlier reported formula [19]:
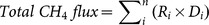
where, R*_i_* is CH_4_ flux (mg m^−2^ day^−1^) in the *i* ^th^ sampling interval, D*i* was the number of days in the *i* ^th^ sampling interval, and n was the number of sampling intervals.

### Chemical analysis of soil and manures

Soil samples were collected before the initiation of experiment from the surface layer (0–15 cm) and after rice harvesting from the whole pot, air-dried, ground, and sieved (<2 mm). The samples were analyzed for pH (1∶5 water extraction), and concentrations of SOC (Walkley and Black method [20]), exchangeable Ca^2+^, Mg^2+^, and K^+^ (1 N ammonium acetate pH 7.0, AA, Shimadzu 660, Kyoto), available phosphate (Olsen method [21]). In addition, soil samples were collected with a core sampler (0–15 cm) to investigate changes of DOC concentrations [22] at important rice seasons such as 30 (maximum tillering), 45 (panicle initiation), 80 (heading), and 120 (harvesting) DAT during rice cultivation. DOC was determined using a procedure described by Jones and Willett [22] with slight modification. Briefly, fresh soil samples were homogenized with deionized water (soil:water = 1:10, w/v basis) by shaking at 120 rpm for 1 hr. After extraction, the suspension was centrifuged at 5000 rpm for 15 minutes, filtered through a 0.45 µm filter, and the supernatant was used for organic carbon determination by Shimadzu total organic carbon analyzer (TOC-VCPN, Shimadzu, Kyoto).

The cattle and swine manure samples were oven-dried at 70°C for 72 h, ground and then analyzed for total C and N by using elemental analyzer (CHNS-932 Analyzer, Leco, St. Joseph, MI). DOC concentrations were determined using the same procedure described as soil DOC measurement.

### DNA extraction from soils and PCR amplification

The soil samples were collected at the same time with DOC analysis to compare the methanogenic abundance and diversity during rice cultivation were immediately lyophilized by Pilot Lyophilizer (PVTFD50A, Ilsin, Korea) and then sieved through 2 mm size. The DNA was extracted from the lyophilized soil samples by using FastDNA SPIN Kit for Soil (MP Biomedical, Santa Ana, CA, USA) following the manufacturer’s instructions. The extracted DNA was used as a template for PCR using suitable primers [23], *mcr*A_forward (5′-GGTGGTGTMGGATTCACACARTAYGCWACAGC-3′) and *mcr*A_reverse (5′-TTCATTGCRTAGTTWGGRTAGTT-3′). The primers were designed to amplify a DNA fragment encoding the *mcr*A gene (the alpha subunit of methyl coenzyme M reductase). The PCR amplification was performed with a Takara Extaq (Takara biotechnology, Japan) using 1 µl of a template (10 ng µl^−1^) in 50 µl of reaction mixture. The PCR amplification was performed with the following reaction conditions: initial denaturation at 95°C for 5 min, 32 cycles of 95°C for 45 s annealing at 55°C for 45 s and 72°C for 45 s, followed by a final extension at 72°C for 5 min. The PCR product was analyzed by electrophoresis on a 1.5% agarose gels and stained with ethidium bromide (EtBr). The target DNA fragment, approximate size of 460–490 bp, was purified using a QIAquick PCR purification kit (Qiagen Sciences, Germantown, MD, USA).

### Cloning, sequencing, and phylogenetic analysis of mcrA genes

One clone library of *mcr*A gene retrieved from 45 DAT samples from cattle manure-applied soils (S1 Figure) with higher CH_4_ emission was generated using the pGEM-T Easy Vector system (Promega, Madison, WI, USA) according to the manufacturer’s instruction. Forty six randomly selected clones were sequenced by a 3730 DNA analyzer (Applied Biosystems, Foster City, California, USA) at the Macrogen sequencing service (Macrogen Inc., Seoul, Korea). Phylogenetic trees were constructed using the neighbor-joining program MEGA5.0 according to the protocol described by Ma et al. [24]. The gene sequences were deposited into the GenBank nucleotide sequence database under accession numbers (KC510418 to KC510463).

### Changes of methanogenic diversity by T-RFLP

Terminal restriction fragment length polymorphism (T-RFLP) analysis was carried out to investigate the changes of methanogenic community in rice paddy soil [25]–[27]. In this study, T-RFLP patterns were compared in control and cattle and swine manure-applied soil at 40 Mg ha^−1^ during rice cultivation. The primers used for the PCR amplification were described above, except that the forward primer was labeled with 6-carboxyfluorescein (6-FAM). The PCR product was purified by a PCR purification kit (Qiagen, Valencia, CA) and then digested by restriction enzyme *Sau96I* [G’GNCC] (New England Biolabs, NEB, Beverly, MA) at 37°C for 3 hr. The terminal restriction fragments (T-RF) were purified with SigmaSpin^TM^ Post-Reaction Clean-Up Columns (Sigma, St. Louis, MO, USA) and analyzed by using an ABI 3730 capillary sequencer (Applied Biosystems, Foster City, CA, USA). The T-RFLP pattern was analyzed with Genescan 3.7 software (Applied Biosystems) using peak height integration of the different T-RFs. The percent fluorescence intensity for single T-RF was calculated by using total fluorescence intensity of T-RFs. The diversity indices of methanogen community were assessed by Shannon diversity index (*H’*) and Shannon evenness (E), which were calculated based on T-RFLP data according to the method of Egert et al. [28].

### Real-time quantitative PCR of mcrA genes

The quantitative PCR of *mcr*A gene copy numbers were analyzed by BioRad CFX96 real-time thermocycler (BioRad Laboratories, Hercules, CA, USA). The reaction mixture (SYBR Green Real-time PCR Master Mix, Toyobo, Japan) was composed of 10 pmol of each primer [23], 1 µl template DNA and sterilized distilled water added to make the final volume up to 50 µl. The amplification was carried out by modifying earlier reported method [13]. The initial denaturation was done at 95°C for 10 min, followed by 40 cycles at 94°C for 45 sec, 52°C for 45 sec and 72°C for 45 sec. The DNA standard was prepared from the purified plasmid DNA of *mcr*A clone after 10-fold serial dilutions of plasmids containing a sequence of *mcr*A gene from *Methanosarcina mazei*. Amplification efficiency of the PCR was calculated using standard curves with the following formula:
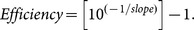



The amplifications of serial diluted standards were performed for samples of each pot to minimize the inhibitory effect exerted by substances co-extracted with DNA. The quality of the amplification was evaluated by the generation of a melting curve for the PCR product.

### Statistical analysis

Statistical analysis was carried out using analytical software SAS 9.1 (SAS Institute Inc., Cary, NC). One way analysis of variance (ANOVA) was carried out to determine significance of treatments. Tukey’s post-hoc test was used to separate treatment means when the F-test showed to be significant at the *P<0.05* probability level. Linear regression analysis was performed to evaluate relationships between response variables. Non-metric multidimensional scaling (NMDS) analysis was performed on T-RFLP profiles of *mcr*A genes to investigate the difference of methanogenic community among the samples by R statistical software (version 2.6.0).

## Results

### Methane emissions

**Figure 1:**
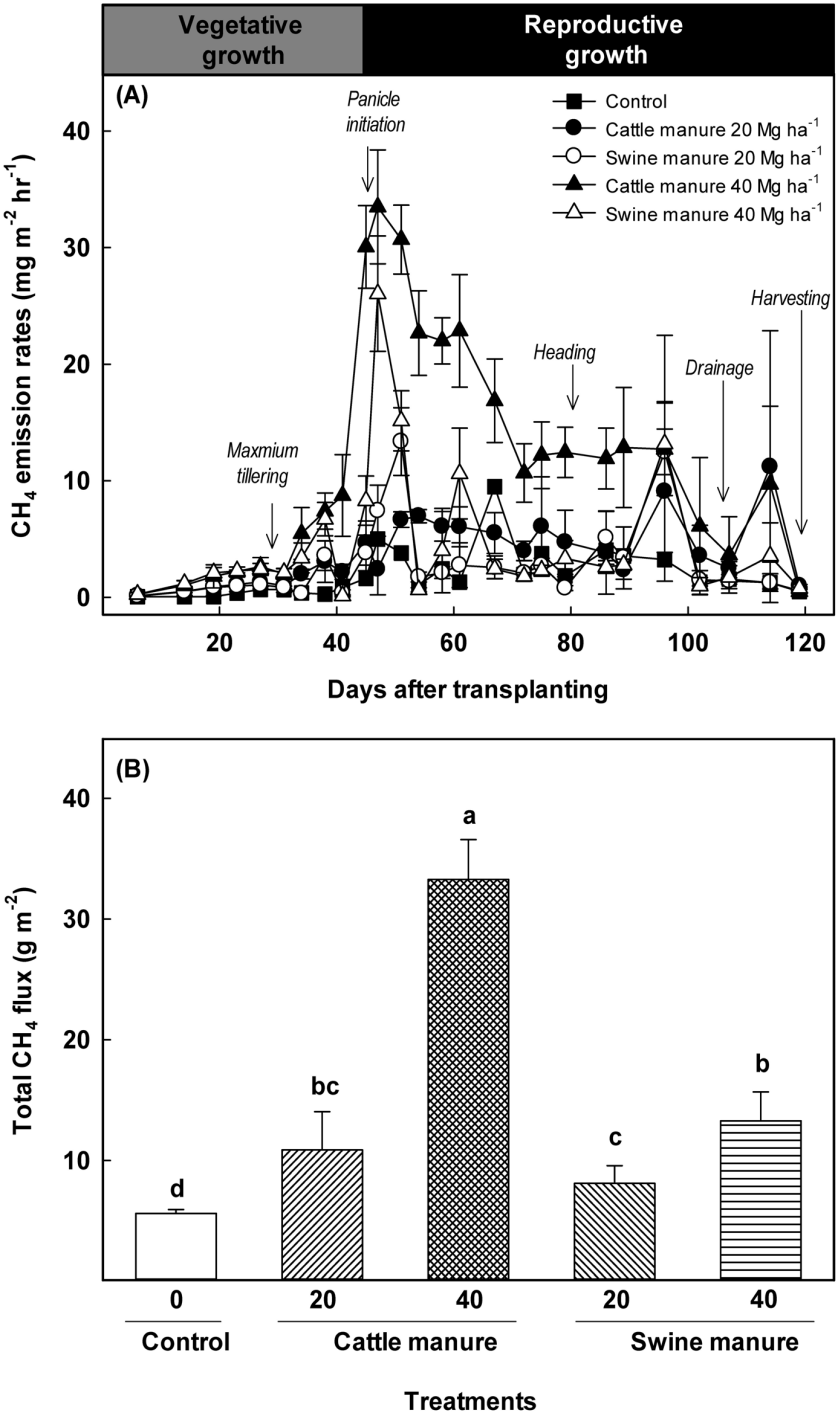
Changes in CH_4_ flux with time (A) and total CH_4_ fluxes (B) under different manure application during rice cultivation. Bars represent standard errors (n = 3). Different letters indicate significant difference (One-way ANOVA, *p<0.05*).

Irrespective of treatments, CH_4_ emissions were low at the initial stage of rice cultivation and gradually increased with plant growth up to the reproductive stage (47 DAT) (Fig. 1A). The highest CH_4_ flux was recorded within the first 45–51 DAT. Thereafter, CH_4_ flux gradually decreased at plant maturity and finally declined to the background level during harvest. Both swine and cattle manure applications significantly increased CH_4_ flux and that increase was proportional to the rates of manure applications especially at the initial stage of rice cultivation (up to 75–80 DAT). Total CH_4_ flux in the control was 5.62 g m^−2^, which was significantly increased by 44–49% (8.11–33.3 g m^−2^) after manures applications (Fig. 1B). The CH_4_ flux from cattle manure was higher than those swine manure (up to 40–80 DAT). These differences in CH_4_ emissions were more pronounced at 40 Mg ha^−1^ cattle manure and swine manure than at other rates.

### Soil and rice growth characteristics

Application of manures significantly increased the levels of SOC, total N, available P_2_O_5,_ and exchangeable K^+^ concentrations as compared to the control (Table 1). In general, cattle manure was more effective in improving soil properties, such as SOC, total N, available P_2_O_5_, and exchangeable K^+^ than swine manure with the same rates of application. The SOC, total N, available P_2_O_5_, and exchangeable K^+^ concentrations were slightly higher in cattle manure-applied soils (9.3–11.4 g kg^−1^, 1.35–1.47 g kg^−1^, 50.3–73.3 mg kg^−1^, and 0.29–0.35 cmol^+^ kg^−1^) than swine manure (8.9–11.1 g kg^−1^, 1.27–1.35 g kg^−1^, 50.1–60.0 mg kg^−1^, and 0.23–0.26 cmol^+^ kg^−1^), although not statistically significant except for the exchangeable K^+^ at 40 Mg ha^−1^ cattle manure-applied soil. On the other hand, manure application significantly decreased exchangeable Ca^2+^ and Mg^2+^ concentrations compared to the control treatment.

**Table 1:** Soil properties and rice growth characteristics at harvesting.

Parameters	Manure application level (Mg ha^−1^)				
	Control	Cattle		Swine	
	0	20	40	20	40
*Soil properties*					
pH (1∶5, H_2_O)	7.0^a^	6.6^b^	6.3^b^	6.7^b^	6.3^b^
SOC (g kg^−1^)	7.9^c^	9.3^b^	11.4^a^	8.9^b^	11.1^a^
Total N (g kg^−1^)	1.23^b^	1.35^ab^	1.47^a^	1.27^ab^	1.35^a^
Available P_2_O_5_ (mg kg^−1^)	43.7^b^	50.3^ab^	73.3^a^	50.1^ab^	60.0^a^
Exchangeable cation (cmol^+^ kg^−1^)					
K^+^	0.12^d^	0.29^ab^	0.35^a^	0.23^bc^	0.26^c^
Ca^2+^	4.97^a^	4.00^c^	4.24^bc^	4.17^b^	4.25^b^
Mg^2+^	0.59^a^	0.51^b^	0.45^bc^	0.48^c^	0.45^c^
*Plant growth characteristics*					
Grain yield (g pot^−1^)	18.3^ab^	21.5^a^	15.6^b^	20.9^a^	15.3^b^
Straw yield (g pot^−1^)	161.7^b^	167.6^b^	189.3^a^	161.8^b^	181.4^a^
Total biomass (g pot^−1^)	180.1^b^	189.2^ab^	204.9^a^	182.7^ab^	196.7^a^

*Values in the same row within same parameters followed by different letters are significantly different at *p*<0.05 according to Tukey’s post-hoc test for the separation of means.

**Figure 2:**
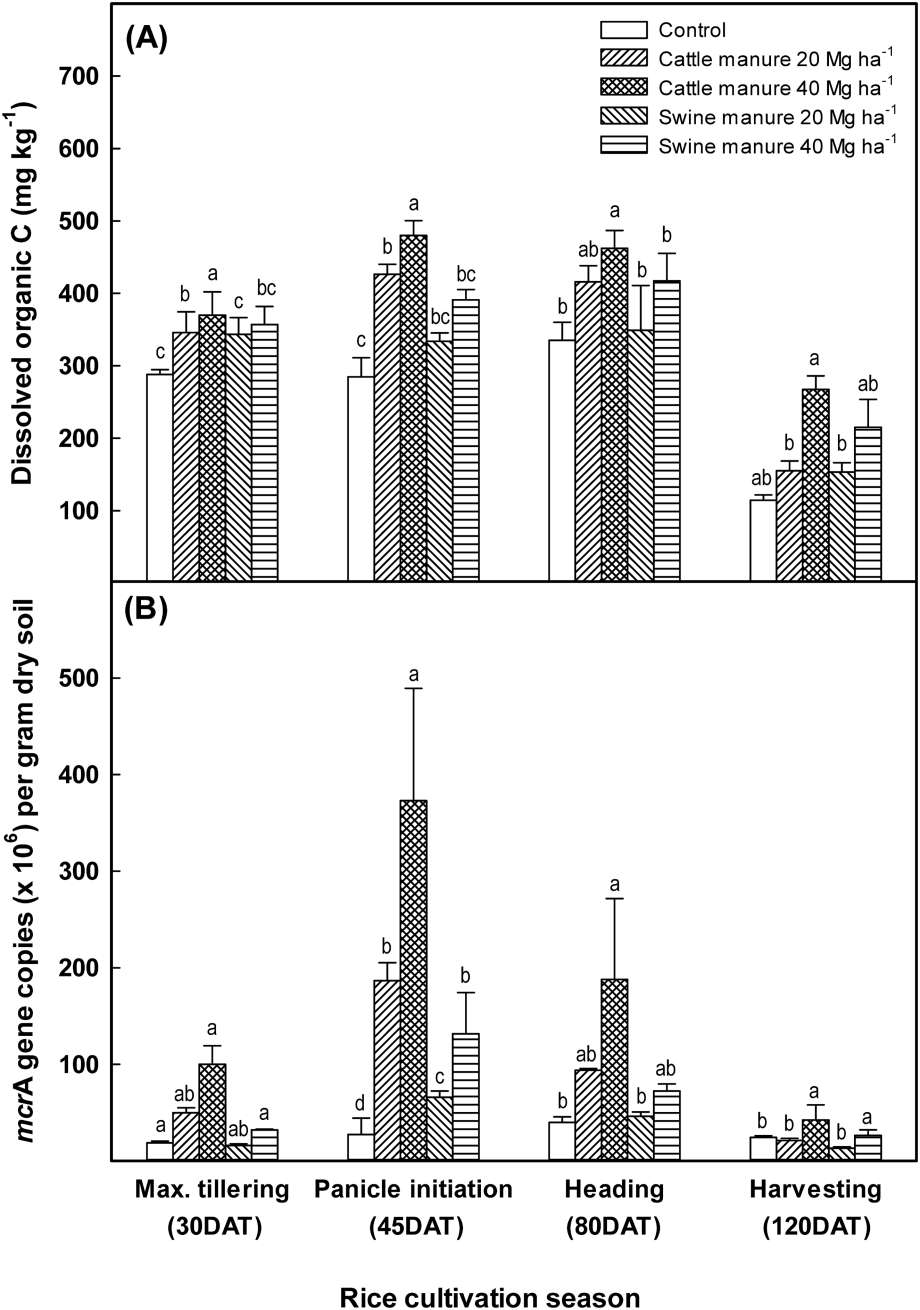
Changes in DOC concentration with time (A) and numbers of total *mcr*A gene copies (B) in soils incorporated with different manures. Bars represent standard errors (n = 3). Different letters at the same stage indicate significant difference (One-way ANOVA, *p<0.05*).

Regardless of treatments, comparatively low DOC contents were observed at the initial stage of rice growth, though the values of DOC concentrations were increased with plant growth. Dissolved organic C concentrations gradually decreased with plant maturation. Cattle and swine manure applications significantly increased DOC concentration and the extent of the increase of DOC in soil was proportional to the manure application rates. Cattle manure, regardless of application rates, was more effective in increasing DOC concentration than the swine manure (Fig. 2A).

The highest level (40 Mg ha^−1^) of manure application significantly increased total biomass of rice plants over the control. Cattle manures produced higher biomass production than swine manure applications, but the difference was not significant (Table 1). Rice grain yield was significantly increased by manure applications at 20 Mg FW ha^−1^ than at 40 Mg ha^−1^, irrespective of the manure treatments.

### Abundance and diversity of methanogens

The *mcr*A gene copy number showed the same trend with the DOC concentrations during rice cultivation (Fig. 2B). Over-all pattern indicated low methanogen abundance at the initial stages of cultivation and increased significantly with plant growth, regardless of the manure applications. The *mcr*A abundance was the highest at the panicle initiation stage (45 DAT). Methanogen abundance increased significantly with manure addition, and the cattle manure-treated soils had higher methanogenic populations than swine manure (Fig. 2B).

**Figure 3:**
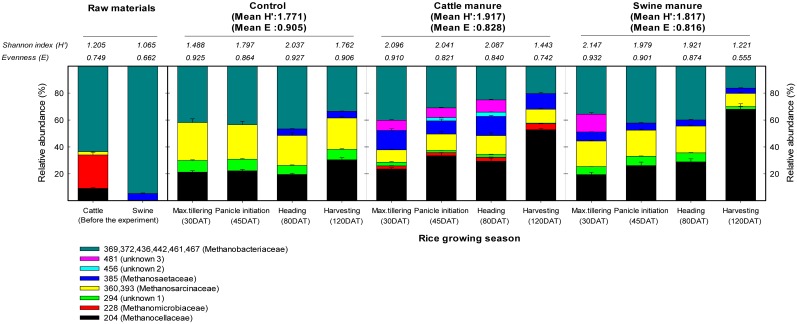
Analysis of methanogen community composition by *mcr*A based T-RFLP under different manure application during rice cultivation. The number indicated Shannon diversity index (*H*).

The phylogenetic analysis revealed that the methanogenic community consisted mainly of *Methanocellaceae* (17.4%), *Methanomicrobiaceae* (8.7%), *Methanosarcinaceae* (30.4%), *Methanosaetaceae* (4.3%), and *Methanobacteriaceae* (39.1%) (S1 Figure and S2 Table). The T-RFLP pattern of *mcr*A genes retrieved from raw manures and soil samples is displayed in Fig. 3. Representatives of *Methanobacteriaceae, Methanomicrobiaceae, Methanocellaceae* and *Methanosarcinaceae* were found in fresh cattle manure, while only two families of methanogens (*Methanobacteriaceae* and *Methanosaetaceae*) were detected in swine manure. Representatives of *Methanobacteriaceae* and *Methanomicrobiaceae* were predominant in cattle manure, while *Methanobacteriaceae* covered ca. 95% of total methanogen population in swine manure.

The families of *Methanobacteriaceae*, *Methanocellaceae, Methanosaetaceae* and *Methanosarcinaceae* were the most predominant in all treatments during the whole rice cultivation period (Fig. 3). The abundance of *Methanosarcinaceae* was comparatively stable throughout the cultivation period in both manure treatments. However, we found that the abundance of *Methanocellaceae* was increased after drainage before rice harvesting but *Methanobacteriaceae* abundance sharply decreased, irrespective of the manure treatments.

The methanogenic communities in manure-applied soils were more complex (mean Shannon diversity index: 1.817 and 1.917) and higher than the control (1.771) (Fig. 3 and S2 Figure). Between the two manure treatments, cattle manure-applied soils contained a more diverse community than the swine manure. The *Methanobacteriaceae*, *Methanocellaceae* and *Methanosarcinaceae* were predominantly present the control treatment (Fig. 3). A more diverse community of methanogens, including *Methanosaetaceae* was observed in the manure-applied soils at the initial rice growing stage (up to 45 DAT) over the control. Methanogenic community slightly differed with swine manure application during rice cultivation. In particular, *Methanosaetaceae* and methanogens of unknown affiliation represented by T-RF 481 bp were observed at the initial stages in swine manure-applied soil, a pattern that did not change much in the control treatment over the experimental period. In contrast, the application of cattle manure significantly increased methanogenic diversity during rice cultivation period than swine manure, except at harvest stage. The *Methanomicrobiaceae, Methanosaetaceae,* and the unidentified T-RF 294 bp and 481 bp were found in cattle manure-applied soils until heading stage, while T-RF 456 bp was only observed at panicle initiation and heading stages. The *Methanomicrobiaceae* (204 bp) and unidentified T-RF 456 bp were only observed in cattle manure treatment, whereas *Methanomicrobiaceae* was observed during whole rice cultivation. However, in swine manure-applied soils, *Methanomicrobiaceae* was not detected which may be due its relative proportion below the detection limit using T-RFLP analysis.

## Discussion

Rice plants can enhance CH_4_ emissions [29] by secreting root exudates enriched in DOC in the soil system [30], [31]. Application of manures, irrespective of the animal originated from, supplied additional nutrients in soil and that in turn increased both straw and total biomass yields of rice plants. It is a well-known fact that CH_4_ emitted from rice paddy soils is transported mostly (60–90%) through the aerenchyma channel of rice plants rather than by molecular diffusion across the water-air interfaces or the release of gas bubbles [32]. Therefore, increased growth of rice can facilitate transport of CH_4_ from the soil to the atmosphere [33] and highly positive correlations between the total CH_4_ flux, and rice straw and biomass yield were observed in this study (Table 2). However, rice grain yield showed negative relationship with total CH_4_ flux. The negative correlation between CH_4_ flux and grain yield has been found by many studies in rice paddy soils [34]–[36]. High level of nitrogen input often caused excessive vegetative growth at the expense of grain yield, thereby reducing grain yield [37], and then might be attributed to comparatively lower yield in 40 Mg manure ha^−1^ treated soils as compared to 20 Mg ha^−1^.

**Table 2:** Correlation between total CH_4_ flux, soil properties and rice yield.

Parameters	Corelation (r) (n = 15)
*Soil properties*	
pH	−0.496
SOC	0.773***
Total N	0.661**
Available P_2_O_5_	0.476
Exchangeable cation	
K^+^	0.495
Ca^2+^	−0.419
Mg^2+^	−0.277
*Plant growth characteristics*	
Grain yield	−0.499*
Straw yield	0.661**
Total biomass	0.611*

*p<0.05, **p<0.01 and ***p<0.001.

**Figure 4:**
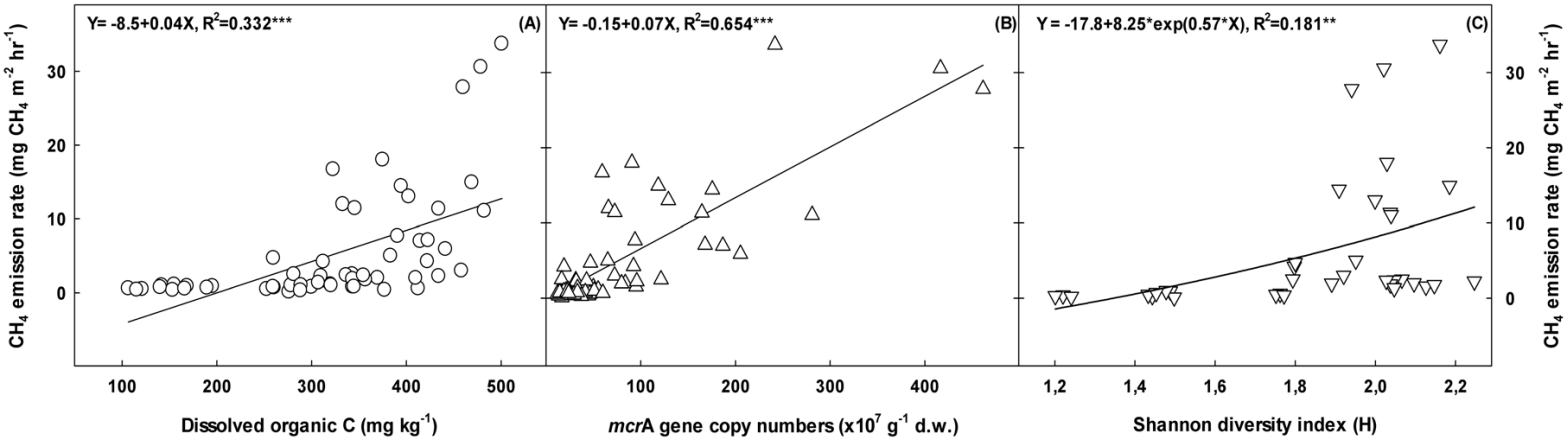
Relationships between CH_4_ emission rate, DOC concentration (A) and *mcr*A gene copy number (B), and Shannon diversity index (C) in soils during rice cultivation.

The availability of energy sources for methanogens, such as labile organic C compounds, rather than total organic C was the most important factor controlling CH_4_ emissions in rice paddy soils [38]–[40]. Although the water-extractable organic C accounted for small proportion of the total SOC in soil [41], it is recognized as one of the most important compounds which may influence microbial proliferation as well as methanogenic activity in soil [40], [42], [43]. Total SOC and DOC concentrations in soil increased significantly with increasing manure application levels (Fig. 2A and Table 1). The highest positive correlation was found between CH_4_ emission rates and DOC concentrations in soil (Fig. 4). The higher labile C concentrations in cattle manure-applied soils might be responsible for the enhanced CH_4_ fluxes as compared to the swine-manure treatments. Carbon and N enrichment can alter biogeochemical C-cycling by affecting the abundance, composition or efficiency of C-cycling taxa [44], [45]. Higher nutrient inputs such as N and P in cattle manure-applied soils enhanced methanogenic substrate availability (S1 Table), which might increase CH_4_ emissions in cattle manure-applied rice paddy soils. The positive correlations between CH_4_ emission rates, and *mcr*A gene copy numbers (R^2^ = 0.654, *p<0.001*) and DOC concentrations (R^2^ = 0.332, *p<0.001*) indicated that activity and abundance of methanogens were mainly controlled by substrate availability (Fig. 4).

The general methanogenic community found in rice paddy soil mainly consisted of *Methanocellaceae*, *Methanosaetaceae*, *Methanosarcinaceae*, and *Methanobacteriaceae* and these were also observed in the other paddy soils [27], [46]. The relative abundance of *Methanosarcinaceae* was comparatively stable during whole rice cultivation in all treatments which may be caused by their ability to utilize diverse group of substrates like H_2_/CO_2_, methanol, or trimethylamine as their energy sources [47]. Drainage before rice harvesting led to increased abundance of *Methanocellaceae* as compared to *Methanobacteriaceae* [48]. This may be due to the ability of representatives of these families to detoxify efficiently reactive oxygen [49].

Methanogens may be broadly classified into two groups based on their requirement for initial organic C compounds and total population and diversity of the methanogen community depend on the amount and quality of applied organic matter. Some methanogen species like *Methanosaetaceae* was only survived when organic matter was incorporated and DOC content was high. Therefore, it could be affirmed that some methanogens can only survive when DOC is abundant and these changes in methanogenic diversity in manure-treated soils are considered as one of the important factors to increase CH_4_ emission during rice cultivation. Interestingly, few methanogens originated from cattle rumens were also survived in soil environment. Though relative activity of these strains was not evaluated during this study, it could be stated that those methanogens possibly were responsible for higher CH_4_ emissions from cattle-manure treated soils as compared to swine manure. Cattle manure contained methanogens probably originated from cattle rumen which survived and persisted in soil. In this study, *Methanomicrobiaceae* were only observed in cattle manure-treated soil during whole rice cultivation which is in line with methanogens being present in cattle manure. Members of the order *Methanomicrobiales* including *Methanomicrobiaceae* use CO_2_ as the electron acceptor and H_2_ as electron donor among hydrogenotrophic methanogens [50]. The energy produced by these reactions is much higher which results in a rapid growth of hydrogenotrophic methanogens than the acetotrophic methanogens [39], [47]. The increased proportion of hydrogenotrophic methanogens might affect more rapid growth in cattle manure-applied soils, which possibly increased CH_4_ emissions in cattle manure-applied soils. Previous reports also showed the presence of *Methanomicrobiaceae* in cattle rumen and cow manure-applied rice paddy field [9], [51]. Apparently cattle manure is a suitable vector to transfer methanogens from digestive systems to other habitats [13], [52], thereby increasing CH_4_ emission from these systems.

Generally, methanogens added to soil will not easily colonize due to the competition with indigenous microorganisms. However, cattle manure can supply high amounts of easily degradable organic matter and nutrients [13], which might help methanogens colonize and adapt to rice paddy ecosystem. In this study, we found that the increase of methanogenic diversity positively correlated (R^2^ = 0.181, *p<0.01*) with the CH_4_ emission rates (Fig. 4). Therefore, this increase of methanogenic diversity by cattle manure application was considered as one of the new factors for highly increased CH_4_ emission during rice cultivation. Based on these findings, a proper pretreatment of cattle manure such as composting which can reduce CH_4_ emissions by up to 50% in compost-applied fields [53] should be considered to reduce CH_4_ emissions and to sustain rice yield during rice cultivation.

## Conclusions

Cattle and swine manure applications improved soil quality. Increased manure rates reduced rice grain yield but enhanced CH_4_ emissions during rice cultivation. However, the greater increase of labile organic C concentrations and plant available nutrients with cattle manure over swine manure enhanced methanogenic abundance in soil suggesting that ruminant methanogens of cattle manure is transferred to rice paddy soils as fresh excrement thereby stimulating more CH_4_ emissions during rice growth. Proper pretreatment of cattle manure should be considered to suppress rumen-based methanogens and CH_4_ emissions and to maintain rice productivity.

## Supporting Information

S1 Figure **Phylogenetic tree of** ***mcr*** **A clone sequences retrieved from 45 DAT cattle manure applied soil.**
(DOCX)

S2 Figure **Non-metric multidimensional scaling (NMDS) analysis of T-RFLP profiles generated from** ***mcr*** **A genes in different manure applied soils during rice cultivation.**
(DOCX)

S1 Table Comparison of nutrient inputs during rice cultivation.
(DOCX)

S2 Table Assignment of T-RFs and analysis of the clone library of *mcr*A clone sequences retrieved from the paddy soil sample on 45 DAT in cattle manure applied soil.
(DOCX)
