# Supplementary material for: Cattle Manure Enhances Methanogens Diversity and Methane Emissions Compared to Swine Manure under Rice Paddy
Source: PLoS One. 2014 Dec 10;9(12):e113593. doi: 10.1371/journal.pone.0113593 (PMC4262209; doi:10.1371/journal.pone.0113593)
Supplement: S1 Figure — Phylogenetic tree of mcr A clone sequences retrieved from 45 DAT cattle manure applied soil. (DOCX) [file pone.0113593.s001.docx]

**
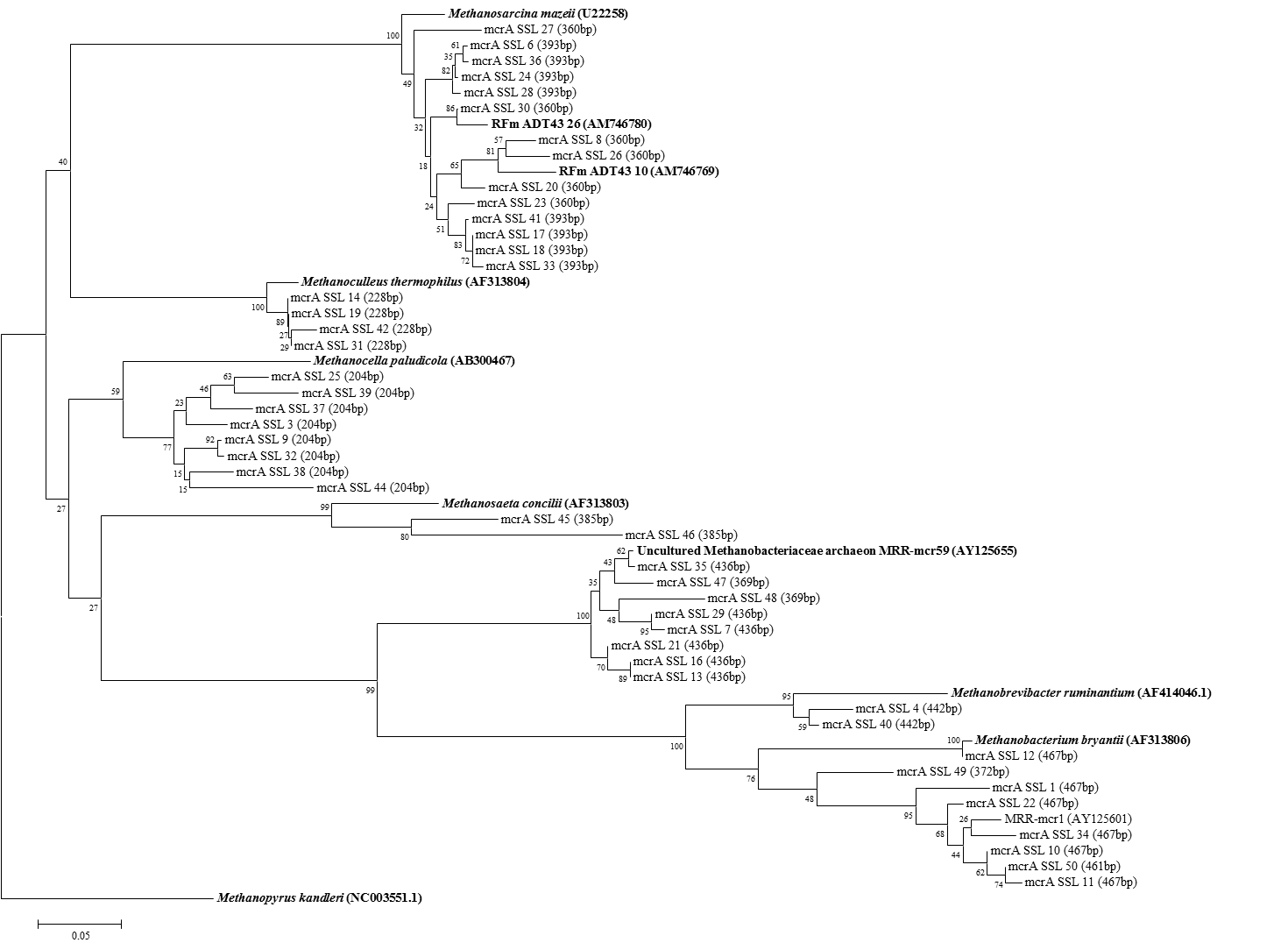
**

**Figure S1.** Phylogenetic tree of *mcr*A clone sequences retrieved from 45 DAT cattle manure applied soil.
