# Supplementary material for: Cattle Manure Enhances Methanogens Diversity and Methane Emissions Compared to Swine Manure under Rice Paddy
Source: PLoS One. 2014 Dec 10;9(12):e113593. doi: 10.1371/journal.pone.0113593 (PMC4262209; doi:10.1371/journal.pone.0113593)
Supplement: S2 Figure — Non-metric multidimensional scaling (NMDS) analysis of T-RFLP profiles generated from mcr A genes in different manure applied soils during rice cultivation. (DOCX) [file pone.0113593.s002.docx]

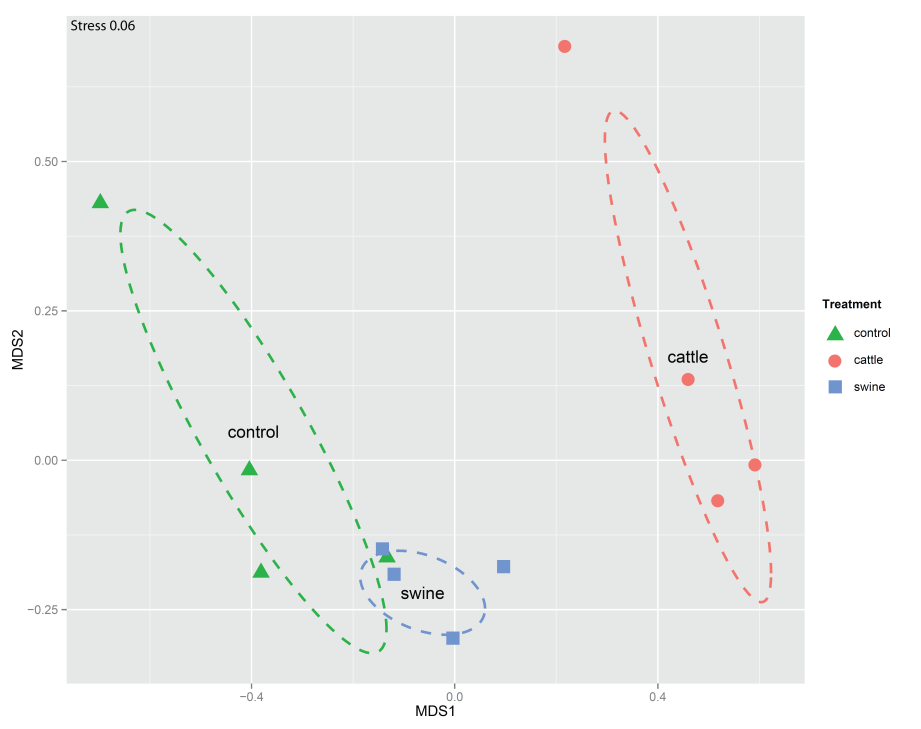


**Figure S2.** Non-metric multidimensional scaling (NMDS) analysis of T-RFLP profiles generated from *mcr*A genes in different manure applied soils during rice cultivation.
