# Supplementary material for: Cattle Manure Enhances Methanogens Diversity and Methane Emissions Compared to Swine Manure under Rice Paddy
Source: PLoS One. 2014 Dec 10;9(12):e113593. doi: 10.1371/journal.pone.0113593 (PMC4262209; doi:10.1371/journal.pone.0113593)
Supplement: S1 Table — Comparison of nutrient inputs during rice cultivation. (DOCX) [file pone.0113593.s003.docx]

**Table S1.** Comparison of nutrient inputs during rice cultivation

| Parameters | Manure application level (Mg ha^-1^) | | | | | | |
| --- | --- | --- | --- | --- | --- | --- | --- |
|  | Control |  | Cattle | |  | Swine | |
|  | 0 |  | 20 | 40 |  | 20 | 40 |
| **Nutrient input (kg ha^-1^)** |  |  |  |  |  |  |  |
| ***Chemical fertilizers (A)*** |  |  |  |  |  |  |  |
| N | 110 |  | 110 | 110 |  | 110 | 110 |
| P_2_O_5_ | 45 |  | 45 | 45 |  | 45 | 45 |
| K_2_O | 58 |  | 58 | 58 |  | 58 | 58 |
| ***Manures (B)*** |  |  |  |  |  |  |  |
| N | 0 |  | 119 | 237 |  | 96 | 193 |
| P_2_O_5_ | 0 |  | 55 | 110 |  | 63 | 127 |
| K_2_O | 0 |  | 8 | 17 |  | 8 | 16 |
| ***Total input (A+B)*** |  |  |  |  |  |  |  |
| N | 110 |  | 229 | 347 |  | 206 | 303 |
| P_2_O_5_ | 45 |  | 100 | 155 |  | 108 | 172 |
| K_2_O | 58 |  | 66 | 75 |  | 66 | 74 |
