# Supplementary material for: Cattle Manure Enhances Methanogens Diversity and Methane Emissions Compared to Swine Manure under Rice Paddy
Source: PLoS One. 2014 Dec 10;9(12):e113593. doi: 10.1371/journal.pone.0113593 (PMC4262209; doi:10.1371/journal.pone.0113593)
Supplement: S2 Table — Assignment of T-RFs and analysis of the clone library of mcrA clone sequences retrieved from the paddy soil sample on 45 DAT in cattle manure applied soil. (DOCX) [file pone.0113593.s004.docx]

**Table S2**. Assignment of T-RFs and analysis of the clone library of *mcr*A clone sequences retrieved from the paddy soil sample on 45 DAT in cattle manure applied soil

| Phylogenetic affiliation of clones | T-RF size (bp)^a^ | Number of clones |
| --- | --- | --- |
| *Methanocellaceae* | 204 | 8 |
| *Methanomicrobiaceae* | 228 | 4 |
| *Methanosarcinaceae* | 360 and 393 | 6 and 8 |
| *Methanosaetaceae* | 385 | 2 |
| *Methanobacteriaceae* | 369,372,436,442,461 and 467 | 2, 1, 6, 2, 1 and 6 |

^a^ The sizes of the T-RFs which related to those determined in the T-RFLP analyses were determined *in silico* for the clone sequences.
